# Crystalline deposits in the cornea and various areas of the kidney as symptoms of an underlying monoclonal gammopathy: a case report

**DOI:** 10.1186/s12882-021-02309-x

**Published:** 2021-04-06

**Authors:** C. Lindemann, P. Enders, P. T. Brinkkoetter, L. A. Völker

**Affiliations:** 1 Department II of Internal Medicine and Center for Molecular Medicine Cologne, (CMMC), University of Cologne, Faculty of Medicine and University Hospital Cologne, Kerpener Str 62, D-50937 Cologne, Germany; 2 Department of Ophthalmology, Medical Faculty and University Hospital of Cologne, University of Cologne, Kerpener Str 62, D-50937 Cologne, Germany; 3 Cologne Cluster of Excellence on Cellular Stress Responses in Ageing-Associated Diseases, Cologne, Germany

**Keywords:** Corneal deposits, Focal segmental glomerulosclerosis, Case report, Monoclonal gammopathy of clinical significance, Monoclonal gammopathy of renal significance, Plasma cell dyscrasias

## Abstract

**Background:**

Plasma cell dyscrasias (PCD) are characterized by an abnormal production of intact monoclonal immunoglobulins or parts such as heavy or light chains. In most cases, the monoclonal protein (also termed paraprotein) is produced by a clonal plasma cell population. The production of monoclonal proteins can result in deposits of various types and localization depending on the type, amount, and electrochemical properties of the paraprotein. One histopathologic presentation, albeit rare, are crystalline deposits. They can form in various organs and hence cause a wide spectrum of symptoms.

**Case presentation:**

A 49-year-old man presented to the emergency department with eyestrain and foreign body sensation after overhead drilling. Examination of the eyes revealed crystalline deposits in the cornea of both eyes. After additional diagnostic testing, deposits were attributed to free light chains. Monoclonal gammopathy of undetermined significance (MGUS) was diagnosed according to serum electrophoresis and immunofixation. Four years later, new onset of proteinuria was detected. A percutaneous biopsy of the kidney showed severe light chain podocytopathy with secondary focal segmental glomerulosclerosis (FSGS) and light chain proximal tubulopathy (LCPT). In these lesions, crystalline deposits identical to the corneal deposits were found in ultrastructural and immunofluorescent analysis. The patient was diagnosed with monoclonal gammopathy of renal significance (MGRS), and a plasma cell directed therapy was initiated.

**Conclusions:**

PCD can present with a wide array of symptoms and are notoriously difficult to diagnose. Extrarenal manifestations such as crystalline deposits in the cornea are one possible manifestation. The case presented herein emphasizes the notion that extrarenal paraprotein deposits warrant a thorough search for the underlying clonal disease.

**Supplementary Information:**

The online version contains supplementary material available at 10.1186/s12882-021-02309-x.

## Background

Plasma cell dyscrasias (PCD) are monoclonal neoplasms which develop from common progenitors in the late B-lymphocyte lineage. Multiple Myeloma (MM) is arguably the most prominent PCD. The most frequent entity, however, is MGUS [1–3]. To distinguish MGUS from MM, criteria have been proposed by the International Myeloma Working Group (IMWG). One important aspect in the differential diagnosis of MGUS and MM is the absence or presence of end organ damage or tissue impairment [4–6]. End organ damage in PCD is defined by hypercalcemia, renal insufficiency, anaemia, bone lesions, and other disorders such as symptomatic hyperviscosity, amyloidosis, and recurrent bacterial infections [4]. Particularly, cast nephropathy and symptomatic hyperviscosity are caused by a high burden of monoclonal protein.

Renal insufficiency in MM is defined by an increase in creatinine above 173 μmol/l or 2 mg/dl or a measured or estimated creatinine clearance below 40 ml/min [6]. The most common cause of renal insufficiency is cast nephropathy, which is characterized by free light chain depositions inside the tubules [7–9]. In addition to this frequent renal manifestation, many other less frequent pathogenic processes have been attributed to monoclonal paraprotein. Renal damage (as indicated by proteinuria, haematuria, leukocyturia, or proximal tubular dysfunction) may occur even without a marked decline in kidney function beyond the diagnostic threshold of multiple myeloma but may still warrant specific therapy. To provide a rationale for treating physicians to initiate a cytotoxic therapy, the entity *monoclonal gammopathy of renal significance* (MGRS) was introduced by the International Kidney and Monoclonal Gammopathy Research Group (IKMG) in 2012 [10, 11]. One rare form of renal involvement in PCD is intracellular crystallization of mostly monoclonal light chains within proximal tubular cells, podocytes, or interstitial histiocytes [12, 13].

Deposits frequently affect the kidneys, and renal abnormalities trigger the work-up that leads to the diagnosis of PCD. Extrarenal manifestations, however, remain underdiagnosed due to their low incidence and frequently subclinical presentation. As early as 2006, Merlini et al. provided an overview of the numerous clinical manifestations of what they termed *dangerous small B-cell clones* and their harmful monoclonal protein products even beyond the kidney [14]. To bring more focus to the numerous, sometimes very rare and diverse manifestations in this context, Fermand et al. introduced the term *monoclonal gammopathy of clinical significance* (MGCS). Here they subsumed all organ damage due to the toxicity of the monoclonal immunoglobulins or other mechanisms in connection with MGUS [15]. However, the concept has not been widely adopted. Corneal depositions of immunoglobulins or free light chains occur in less than 1% of patients with PCD [16, 17]. Due to the low incidence, no information on factors determining risk, progression, and prognosis have been published. PCDs can produce IgG kappa or IgM kappa (or lambda) light chains but deposits consist in kappa (or lambda) light chains alone [16]. Patients usually present with a decrease in visual acuity, glare, blurring, and optical aberrations [18]. In slit-lamp biomicroscopy, corneal deposits can be seen as fine iridescent crystalline deposits in the superficial epithelium and interspersed through the corneal stroma especially in the anterior half [9, 19]. Ophthalmologic findings may mimic deposits seen in cystinosis. Confocal microscopy of the cornea may help to differentiate crystals formed by light chains from those consisting of cysteine. However, final diagnosis of cystinosis or PCD as underlying cause for the corneal crystal deposits is based on the non-ocular comprehensive laboratory work-up.

## Case presentation

**Fig. 1:**
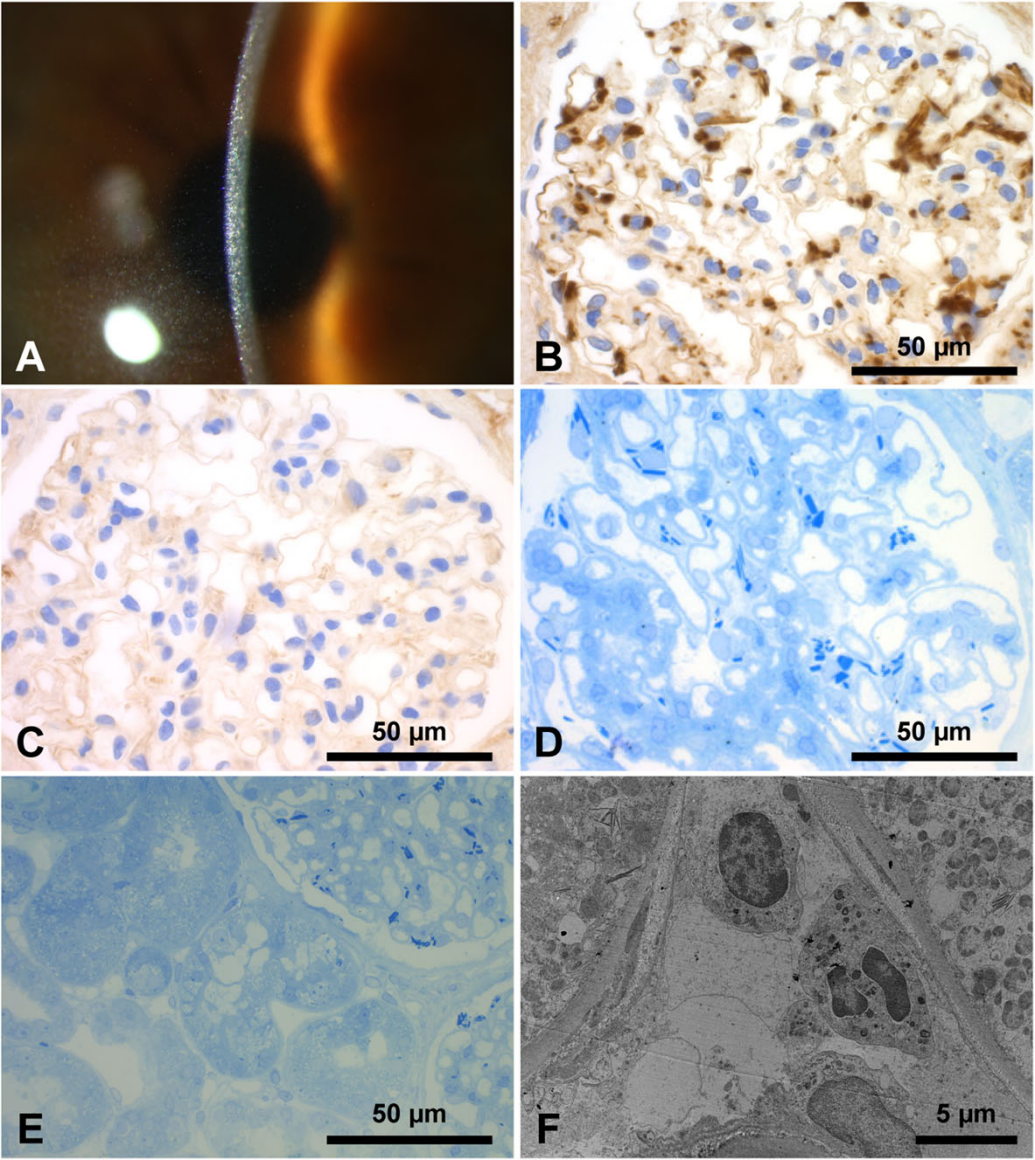
Slit lamp examination shows diffuse intracorneal crystalline deposits (**a**); immunohistochemistry staining for kappa light chains shows positivity of the crystals in the podocytes (**b**) with lambda negativity (**c**); glomerular staining with methylene-azure-blue stains light chain crystals strongly blue with a crystalline appearance (**d**); tubular staining with methylene-azure-blue shows scarce crystalline inclusions in the cytoplasm of proximal tubular epithelial cells (**e**); transmission electron microscopy confirms these crystalline inclusions diagnostic for minimal proximal light chain tubulopathy as intralysosomal (**f**)

A 49-year-old male patient presented to the emergency room with eyestrain and a foreign body sensation in the eye after overhead drilling without wearing protective glasses. The patient suspected a corneal foreign body. During the eye examination conjunctival hyperemia in the right eye was observed as well as corneal deposits in both eyes (Fig. 1a). No subjective visual impairment was reported nor any known ophthalmologic disorders or eye injuries in the past. The patient reported hypothyroidism, which was treated with L-thyroxine. He was on no additional medication, and apart from hypothyroidism, the medical history was unremarkable. Particularly, no weight loss or chronic infections were reported. Serum electrolytes, liver enzymes, hemoglobin concentration, platelets and white blood cell counts, lactate dehydrogenase as well as renal function as determined by the estimated glomerular filtration rate and serum creatinine levels were normal. Urine dipstick, sediment, and proteinuria were not performed initially. Cystinosis, which often presents with corneal deposits, could be ruled out via measurement of cystine content of peripheral blood leukocytes, which was only slightly elevated at 0,3 μmol/g protein (< 0,2 μmol/g protein). A molecular analysis showed no mutation in the CTNS-gene. Serum electrophoresis revealed a mildly elevated gamma globulin fraction of 19% (11.1 – 18.8%) with an extra peak within the fraction. For a more detailed evaluation, an immunofixation of the serum and urine and a serum free light chain assay were ordered. Immunoglobulins G1, G2, G3 in the serum were elevated as well as kappa light chains with 28.6 mg/l (3.3–19.4 mg/l) with a pathologic kappa/lambda ratio of 2.38 (0.26–1.65; range for normal renal function). Bone-lesions were ruled out by whole-body computed tomography. In a synopsis of these findings, MGUS was diagnosed. No bone marrow biopsy was performed at this time so that a differentiation between smoldering multiple myeloma (SMM) and MGUS could not be made. The patient was discharged from the hospital. Regular hematologic follow-ups were scheduled.

Four years later the patient was admitted with increasing proteinuria on a regular follow-up visit. He reported no additional symptoms, particularly, no edema. Renal function and serum electrolytes did not show any relevant abnormalities (see Supplemental Table 1). Free kappa light chains had increased compared to measurements 4 years ago to 60.6 mg/l (3.3–19.4 mg/l) with a pathologic ratio kappa/lambda of now 4.01 (0.26–1.65). Proteinuria of 1203 mg/g creatinine (< 70 mg/g creatinine) with albuminuria of 972 mg/g creatinine (< 20 mg/g creatinine) was present. An elaborate workup of a potential Fanconi syndrome was not conducted, dip stick analysis, however, ruled out overt glycosuria, and there was no hypophosphatemia, hypouricemia, nor features consistent with renal tubular acidosis. On ultrasound examination, the kidneys were of normal size with regular parenchymal structure and unobstructed. To elucidate the cause of proteinuria, an ultrasound-guided transcutaneous renal biopsy was performed. Pathologic examination revealed severe light chain podocytopathy, focal and segmental glomerulosclerosis (FSGS), and minor light chain proximal tubulopathy (LCPT). Immunohistochemistry showed positivity for kappa light chains (Fig. 1b) without amyloidosis-type amorphous deposits upon Congo red staining. There was no staining for lambda light chains (Fig. 1c). Podocytopathy was confirmed by staining with methylene-azure-blue (Fig. 1d). Proximal light chain tubulopathy was confirmed by scarce crystalline inclusions like those much abundant in the podocytes. They were visible in the semi-thin sections stained with methylene-azure-blue (Fig. 1e) and confirmed by electron microscopy as intralysosomal crystals (Fig. 1f). No fibrils were seen. FSGS was judged to be secondary to the light chain podocytopathy.

During the course of the disease, bone marrow biopsy was performed and revealed 10% bone marrow plasma cells. According to the revised 2017 IKMG criteria, smouldering myeloma with renal complications as in this case should be classified as MRGS to justify cytotoxic therapy [20]. First and second line therapies with bortezomib/dexamethasone and lenalidomide/dexamethasone did not achieve a lasting reduction in proteinuria while free kappa light chains remained fairly low. Ultimately, lytic bone lesions were detected on a routine CAT-scan, and multiple myeloma was diagnosed. The patient is currently scheduled for high dose chemotherapy followed by autologous stem cell transplantation.

## Discussion and conclusions

**Fig. 2:**
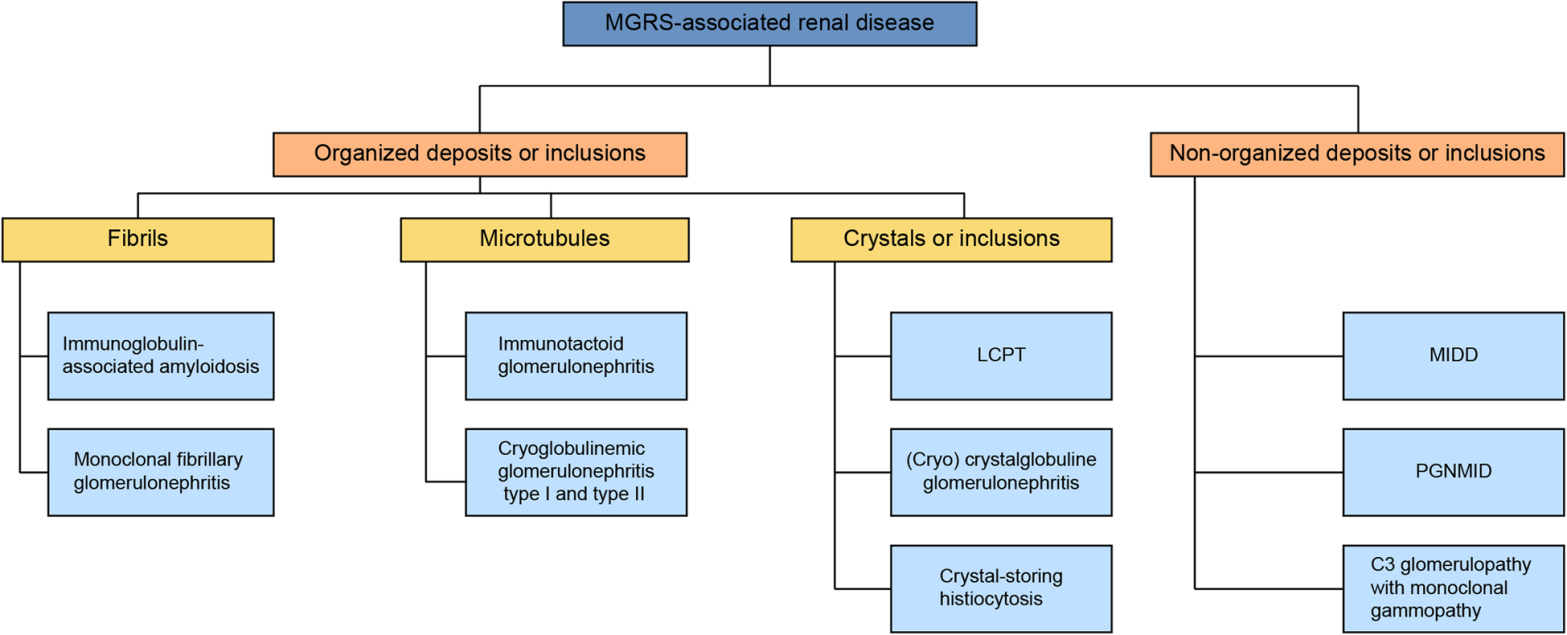
Diagram of MGRS-associated renal diseases based on the classification scheme proposed in 2017 by the International Kidney and Monoclonal Gammopathy Research Group (IKMG) modified from [11]

PCD may present with various organ manifestations. Some end-organ markers are used to define MM and distinguish it from lower-grade PCDs such as MGRS/MGUS [4, 6]. Organ deposits formed by whole or parts of monoclonal immunoglobulins occur frequently. Particularly, renal deposits have been at the center of clinical and scientific interest as they conveniently showcase the pathophysiological mechanisms of deposition and subsequent organ damage and may constitute an indication for therapy independent of the diagnosis of multiple myeloma. To ensure a correct classification of the renal deposits, a renal biopsy and a thorough pathologic workup including light microscopy, immunohistochemistry, and transmission electron microscopy studies in combination with the patient’s medical history and laboratory is needed [20]. Differences in histopathologic findings are driven by the properties of the paraprotein. As shown in a mouse model, specific light chains in the kidney will always form the same type of deposit such as tubular casts, basement-membrane precipitates, or crystals [21]. In addition, it has also been shown that the pathogenicity of a specific light chain is similar in humans and rodents so that structural features of a light chain that lead to nephrotoxicity are independent of species. Although several pathogenetic relationships have been demonstrated using animal models, it should be noted that there are still many unresolved issues, such as the establishment of a suitable model for the study of AL amyloidosis [22]. Transmission electron microscopy is crucial to determine the structure of the deposits. A first differentiation is made between non-organized and organized deposits, which show a specific substructure. Non-organized deposits are found in patients with monoclonal immunoglobulin deposition disease (MIDD) and proliferative glomerulonephritis with monoclonal immunoglobulin deposits (PGNMID). Organized deposits can be further divided into fibrillar, microtubular, or crystalline/inclusionary forms. Crystalline or inclusionary forms manifest as LCPT, crystal storing histiocytosis (CSH), and (cryo) crystalglobulin glomerulonephritis (Fig. 2) [20, 23]. In a significant number of patients with LCPT, the clinical picture of Fanconi syndrome with symptoms such as normoglycemic glycosuria, metabolic acidosis, hypophosphatemia, hypouricacidemia and aminoaciduria may also occur [24, 25].

The underlying podocytopathy is caused by light chains forming intracellular crystal deposits. Interestingly in our patient, crystalline deposits manifested in a second renal structure, the proximal tubules, causing LCPT. Kappa light chain restriction is predominantly found in LCPT in line with our findings [26]. LCPT has also been described without crystals but in a smaller percentage of cases. This entity shows predominantly lambda light chain restriction [12]. Similar cases have already been described, some of which already fulfilled the criteria for MM or in which Fanconi syndrome was present [27, 28]. FSGS reported in our case is a rare renal disorder in PCD. Only a small number of case studies report FSGS in conjunction with PCD. FSGS in these cases is most likely secondary to paraprotein-associated podocytopathies, and causality remains to be demonstrated. Although considered secondary, clinical symptoms of FSGS with underlying PCD may vary: Our patient showed proteinuria in the sub-nephrotic range without edema or hypalbuminemia. In contrast, other cases have been reported in which a full nephrotic syndrome was evident with pronounced proteinuria, edema, and hypalbuminemia [28–31]. In summary, the clinical picture of FSGS associated with PCD shows a wide range from only moderate proteinuria to full nephrotic syndrome, which should always be taken into account in diagnosis.

Extrarenal crystalline deposits in PCD are quite rare and may affect the cornea [9, 16, 19, 32]. Symptoms in patients with corneal deposits are variable and range from none to strong visual impairment [16]. There is a number of mechanisms causing corneal deposits such as transport via the tear film, diffusion from aqueous fluids, and influx via the perilimbal vascular arcades [33, 34]. Occasionally, as in the case presented herein, corneal involvement is the first organ manifestation which suggests the diagnosis of PCD [35]. As ocular symptoms may precede the diagnose of MGUS by years, it is crucial to consider the possibility of PCD if crystalline corneal deposits are detected. Excluding cystinosis, another entity with crystalline deposits, should be part of this workup. The eyes should be additionally examined with in vivo confocal microscopy (IVCM) or corneal densitometry to detect crystalline deposits if present [17, 18]. The clinical workup should include a thorough clinical examination, and serum and urine electrophoresis, immunofixation, and quantification of serum free light chains and -ratios to rule out any PCD. Additional diagnostic modalities such as CAT- or MR-scan or bone marrow biopsies may be necessary.

Due to their low incidence and highly variable presentation, only few studies on the treatment of crystal deposition in PCD have been published. Mainly, a plasma cell directed therapy to reduce the amount of the involved light chain is recommended using similar regimens as in MM [26]. The introduction of the concept of MGCS by Fermend et al. was an important step to bring organ threatening manifestations of monoclonal gammopathies outside the kidney to the limelight [15]. To prevent organ loss, physicians need to enter a process of shared decision making with their patients and discuss potentially side effect-prone therapies in an off-label indication. Optimal regimens in these situations remain elusive, and effects can only be extrapolated from myeloma trials with due caution. Therefore a thorough workup is crucial to exclude MGRS, MM, and other paraprotein-producing hematologic malignancies and to confirm MGCS. It appears prudent to base therapy modalities on parameters such as paraprotein burden, imminence of organ function loss, side effect profile, kidney and liver function, and ultimately patient preferences.

In conclusion, extrarenal manifestations in PCD as seen in our patient can become organ-threatening and have a significant impact on quality of life. There is little data supporting the use of cytotoxic therapeutic regimens in these patients. However, they may be needed in some patients to prevent organ-function loss. Medical subspecialists beyond hematologist and nephrologist should be aware of rare manifestations of PCD in their scope of expertise and should refer for a thorough workup for paraprotein-producing (pre)-malignant cell clones.

## Supplementary Information

**Additional file 1.**

## Data Availability

The datasets used during the report are available from the corresponding author on reasonable request.

## Abbreviations

CSH: Crystal storing histiocytosis
FSGS: Focal segmental glomerulosclerosis
IMWG: The International Myeloma Working Group
IVCM: In vivo confocal microscopy
LCPT: Light chain proximal tubulopathy
MGRS: Monoclonal gammopathy of renal significance
MGUS: Monoclonal gammopathy of undetermined significance
MIDD: Monoclonal immunoglobulin deposition disease
MM: Multiple myeloma
PCD: Plasma cell dyscrasias
PGNMID: Proliferative glomerulonephritis with monoclonal immunoglobulin deposits
SMM: Smoldering multiple myeloma
